# A back propagation neural network approach to estimate the glomerular filtration rate in an older population

**DOI:** 10.1186/s12877-023-04027-5

**Published:** 2023-05-24

**Authors:** Shimin Jiang, Yetong Li, Yuanyuan Jiao, Danyang Zhang, Ying Wang, Wenge Li

**Affiliations:** 1 Department of Nephrology, China-Japan Friendship Hospital, No. 2 East Yinghuayuan Street, Chaoyang District, Beijing, 100029 China; 2 Department of Nephrology, Beijing Children’s Hospital, National Center for Children’s Health, Capital Medical University, Beijing, 100045 China; 3 Graduate School of Peking Union Medical College, Peking Union Medical College and Chinese Academy of Medical Sciences, Beijing, 100730 China

**Keywords:** Elderly, Glomerular filtration rate, Estimation equation, Back propagation neural network, Serum creatinine

## Abstract

**Background:**

The use of creatinine-based glomerular filtration rate (GFR)-estimating equations to evaluate kidney function in elderly individuals does not appear to offer any performance advantages. We therefore aimed to develop an accurate GFR-estimating tool for this age group.

**Methods:**

Adults aged ≥ 65 years who underwent GFR measurement by technetium-99 m-diethylene triamine pentaacetic acid (^99m^Tc-DTPA) renal dynamic imaging were included. Data were randomly split into a training set containing 80% of the participants and a test set containing the remaining 20% of the subjects. The Back propagation neural network (BPNN) approach was used to derive a novel GFR estimation tool; then we compared the performance of the BPNN tool with six creatinine-based equations (Chronic Kidney Disease-Epidemiology Collaboration [CKD-EPI], European Kidney Function Consortium [EKFC], Berlin Initiative Study-1 [BIS1], Lund-Malmö Revised [LMR], Asian modified CKD-EPI, and Modification of Diet in Renal Disease [MDRD]) in the test cohort. Three equation performance criteria were considered: bias (difference between measured GFR and estimated GFR), precision (interquartile range [IQR] of the median difference), and accuracy P30 (percentage of GFR estimates that are within 30% of measured GFR).

**Results:**

The study included 1,222 older adults. The mean age of both the training cohort (n = 978) and the test cohort (n = 244) was 72 ± 6 years, with 544 (55.6%) and 129 (52.9%) males, respectively. The median bias of BPNN was 2.06 ml/min/1.73 m^2^, which was smaller than that of LMR (4.59 ml/min/1.73 m^2^; p = 0.03), and higher than that of the Asian modified CKD-EPI (-1.43 ml/min/1.73 m^2^; p = 0.02). The median bias between BPNN and each of CKD-EPI (2.19 ml/min/1.73 m^2^; p = 0.31), EKFC (-1.41 ml/min/1.73 m^2^; p = 0.26), BIS1 (0.64 ml/min/1.73 m^2^; p = 0.99), and MDRD (1.11 ml/min/1.73 m^2^; p = 0.45) was not significant. However, the BPNN had the highest precision IQR (14.31 ml/min/1.73 m^2^) and the greatest accuracy P30 among all equations (78.28%). At measured GFR < 45 ml/min/1.73 m^2^, the BPNN has highest accuracy P30 (70.69%), and highest precision IQR (12.46 ml/min/1.73 m^2^). The biases of BPNN and BIS1 equations were similar (0.74 [-1.55−2.78] and 0.24 [-2.58−1.61], respectively), smaller than any other equation.

**Conclusions:**

The novel BPNN tool is more accurate than the currently available creatinine-based GFR estimation equations in an older population and could be recommended for routine clinical use.

**Supplementary Information:**

The online version contains supplementary material available at 10.1186/s12877-023-04027-5.

## Highlights

Accurate estimation of kidney function in older adults is an important step to management of chronic kidney disease.

BPNN tool highly improved precision and accuracy, although bias remains suboptimal.

BPNN tool can be recommended for clinical use routinely, especially for patients with GFR < 45 ml/min/1.73 m^2^.

**Supplementary Information:**

The online version contains supplementary material available at 10.1186/s12877-023-04027-5.

## Introduction

Glomerular filtration rate (GFR) is regarded as the best overall index of kidney function in health and disease. Accurately estimating the GFR is an important step in the diagnosis, classification and management of chronic kidney disease (CKD) [1], especially in persons older than 65 years with CKD. This is because, in this age group, delayed referrals for the management of CKD may lead to suboptimal outcomes, including increased mortality, increased hospitalization rates, and increased referrals for renal replacement therapy.

In 2009, a new equation based on serum creatinine was developed by the CKD-Epidemiology Collaboration (CKD-EPI) [2], which was proven to be more accurate than the Modification of Diet in Renal Disease (MDRD) Study equation in those with a GFR ≥ 60 ml/min/1.73 m^2^ [2–4]. Thereafter, a new Asian modified CKD-EPI equation was developed from the general population, which might be a better predictor of the GFR than the original equation in Chinese individuals [5]. Considering that ageing correlates with structural and physiological changes in kidney and muscle mass, this may affect the estimation of the GFR based on serum creatinine. Thus, there are concerns about the accuracy of the GFR-estimating equation in older adults; this has led to proposals for new equations, including the Lund-Malmö Study Revised (LMR) [6], Berlin Initiative Study-1 (BIS1) [7], full age spectrum (FAS) [8], and modified FAS (i.e., European Kidney Function Consortium, EKFC) [9] equations. However, to date, there do not appear to be performance advantages for the use of any of these equations in persons aged 65 years and older [10, 11].

In recent years, the application of machine learning in health care, especially deep learning, has been proposed to extract information from large datasets [12, 13]. Deep learning combined with human intelligence can help clinicians provide better care for patients and improve personal health. Back propagation neural networks (BPNNs) represent one of the most notable advances in deep learning. We therefore attempted to develop a more accurate GFR-estimating model for this age group using the BPNN approach, tested this model, and compared its performance with other creatinine-based GFR-estimating equations (e.g., the CKD-EPI, BIS1, EKFC, LMR, and MDRD[14]).

## Materials and methods

### Study subjects

**Fig. 1:**
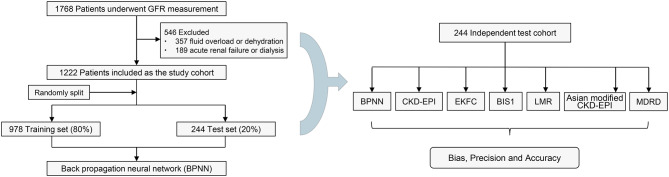
Flowchart of the study. GFR, glomerular filtration rate; CKD-EPI, Chronic Kidney Disease-Epidemiology Collaboration equation; EKFC, European Kidney Function Consortium equation; BIS1, Berlin Initiative Study-1 equation; LMR, Lund-Malmö Revised equation; MDRD, Modification of Diet in Renal Disease equation

This retrospective study was planned to include adults aged 65 years and older who underwent a GFR measurement by technetium-99 m-diethylene triamine pentaacetic acid (^99m^Tc-DTPA) renal dynamic imaging at our centre (May 2011 and May 2022). Participants diagnosed with acute kidney failure, receiving dialysis, or suffering from dehydration or fluid overload at the time of GFR measurements were excluded. Figure 1 displays a screening flowchart of the participants in the present study.

Data from the study cohort were randomly split into a training set containing 80% of the subjects and a test set containing the remaining 20% of subjects. The BPNN model was developed in the training set and tested in the independent test set.

### Clinical and laboratory data

The collected information included age, sex, height, weight, GFR, serum creatinine concentration, and presence of diabetes. Diabetes was diagnosed according to the 2022 American Diabetes Association (ADA) criteria [15]. Serum creatinine levels were determined using an enzymatic sarcosine oxidase method under fasting conditions with a Beckman AU5800 biochemical analyser (Beckman Coulter, Inc., Brea, CA). The detection of GFR was performed by the ^99m^Tc-DTPA renal dynamic imaging method [16]. The results were normalized to a body surface area (BSA) of 1.73 m^2^, as described by the Dubois method [17]. BSA (m^2^) = 0.007184 × body weight (kg)^0.425^ × body height (cm)^0.725^.

### BPNN model

In the present study, we developed a novel BPNN model for GFR estimation using a combination of four independent variables including age, sex, serum creatinine and diabetes. Before the BPNN model was established, data preprocessing on the training cohort was performed. The continuous variables (age and serum creatinine) were log-transformed, and then these four variables were normalized so that the values of all features were distributed in the range of 0–1. This model is composed of 2 hidden layers with 2 and 1 neurons in each layer. The number of neurons in the input layer corresponded to the four independent variables, while the number of neurons in the output layer was just 1, which corresponded to the dependent variable (measured GFR). The activation function ReLU (rectified linear unit) and the Adam optimizer were used with 100 epochs (number of learning cycles). The batch size for each training iteration was set to 5. Initially, the biases of each neuron and weights between layers were initialized randomly according to the normal distribution. The learning rate was set to 0.1, and no learning rate decay. The mean absolute error (MAE) and R squared were calculated in the test cohort as performance metrics for the regression model. The smaller the value of the MAE and the higher the value of R squared, the better the accuracy with which the model describes the estimated GFR. The whole development of BPNN was implemented by the machine learning software of PyCharm community edition, based on Python language (version 3.6.7, Python Software Foundation).

### Creatinine-based GFR-estimating equations

Six creatinine-based equations were used in the study population for estimating GFR, including the CKD-EPI [2], BIS1 [7], EKFC [9], LMR [6], new Asian modified CKD‑EPI [5], and MDRD [14] equations (Table S1).

### Statistical analysis and model evaluation

Continuous variables were expressed as the mean and standard deviation (SD) or median (interquartile range, IQR). Categorical variables were presented as count (n) and percent frequency (%). Three criteria were considered when evaluating and comparing the performance of the BPNN model and four other equations in the test cohort: bias, precision, and accuracy. Bias was expressed as the median difference between the measured GFR and estimated GFR. Precision was expressed as the IQR of the difference between measured GFR and estimated GFR. Accuracy was defined as the percentage of estimates within 30% of the measured GFR (P30). According to the K/DOQI guidelines, a P30 value ≥ 75% is sufficient for making good clinical decisions [18]. The 95% confidence intervals (CIs) around bias, precision, and P30 values were calculated using a bootstrap method (1000 bootstraps) [19].

Mood’s median test was used to compare the median biases [20]. If Mood’s median test is different, a post-hoc analysis is performed to determine which groups differ from each other group. The difference in P30 between the two equations was determined using Cochran Q with pairwise McNemar’s test and Holm-Bonferroni correction [21, 22]. The Lin’s concordance correlation coefficient (CCC) was used to assess the strength of agreement between each estimated GFR and measured GFR [23]. CCC is a measure of agreement that adjusts the Pearson correlation coefficient downward whenever there is a systematic bias between the methods being compared. A CCC > 0.9 denotes good concordance between the two measurements, a CCC of 0 reflects no concordance at all. Statistical analyses were performed using SPSS (version 27.0; IBM Corp., Armonk, NY, USA) and R (version 4.0.5; Foundation for Statistical Computing, Vienna, Austria; http://www.R-project.org) software. All tests were 2-sided, with p < 0.05 indicating statistical significance.

## Results

### Patient characteristics

Of the initial 1,768 participants, 1,222 met the study criteria (Fig. 1). The main characteristics of eligible participants in the study cohort and the test cohort are shown in Table 1. In the study cohort of participants, the mean (SD) age was 72 (6) years. Among these participants, 544 (55.62%) were male, 461 (47.14%) were living with diabetes, and 727 (74.33%) had CKD. The median (IQR) measured GFR was 49.25 (34.79–66.94) ml/min/1.73 m^2^, and 43 (19.12%) of measurements had GFR values less than 30 ml/min/1.73 m^2^.

**Table 1:** Main characteristics of older adults in the training cohort and the test cohort

Characteristics	Training cohort (n = 978)	Test cohort (n = 244)
Age, years	71.8 ± 6.1	71.9 ± 6.3
Males, n (%)	544 (55.62)	129 (52.87)
BSA, m^2^	1.7 (1.6–1.9)	1.7 (1.5–1.8)
BMI, kg/m^2^	24.9 (22.9−27.7)	24.6 (22.2−27.5)
Serum creatinine, µmol/L	110.15 (80.0–165.0)	116.3 (78.42–173.85)
Diabetes, n (%)	461 (47.14)	121 (49.59)
CKD, n (%)	727 (74.33)	187 (76.64)
Causes of CKD, n (%)		
Chronic glomerulonephritis	218 (30.0)	59 (31.6)
Diabetic nephropathy	145 (19.9)	46 (24.6)
Hypertensive kidney damage	72 (9.9)	20 (10.7)
Kidney tumors	112 (15.4)	19 (10.2)
Unkown	180 (24.7)	42 (23.0)
Measured GFR, ml/min/1.73 m^2^	49.25 (34.79–66.94)	46.81 (32.28–65.60)
≥ 90 ml/min/1.73 m^2^, n (%)	47 (4.81)	15 (6.15)
60–89 ml/min/1.73 m^2^, n (%)	276 (28.22)	61 (25.0)
30–59 ml/min/1.73 m^2^, n (%)	468 (47.85)	113 (46.31)
15–29 ml/min/1.73 m^2^, n (%)	144 (14.72)	47 (19.26)
< 15 ml/min/1.73 m^2^, n (%)	43 (4.4)	8 (3.28)

BSA, body surface area; CKD, chronic kidney disease; GFR, glomerular filtration rate

Data are expressed as mean ± standard deviation, median (interquartile range), and percent frequency

In the test cohort, the mean (SD) age was also 72 (6) years, and 129 (52.87%) were male. Of these participants, 121 (49.59%) had diabetes and 187 (76.64%) had CKD. The median (IQR) measured GFR was 46.81 (32.28–65.6) ml/min/1.73 m^2^. According to the level of measured GFR, 55 (20.54%) of these participants had a GFR below 30 ml/min/1.73 m^2^.

### Establishment of the BPNN tool in the study cohort

We proposed a BPNN model with four independent variables for GFR estimation, including age, sex (males or females), serum creatinine, and diabetes (present or absent). As the BPNN model was complicated, we therefore provided an excel file (Table S2) to implement this model.

To provide a good performance of the estimation model, our study cohort was randomly divided into two subsets: training and test sets. The training set included 978 individuals (80% of the study cohort) and the test set included 244 individuals (20% of the study cohort). The four variables of specific interest were used in both sets (Table 1). The MAE and R squared of the BPNN were 8.81 ml/min/1.73 m^2^ and 0.75, respectively, in the test set from the study cohort.

### Comparisons of performance of the BPNN tool with the EKFC, BIS1, CKD-EPI, LMR, Asian modified CKD-EPI, and MDRD equations in the test cohort

Table 2 shows the performance of the seven equations in the test cohort, determined by calculating the bias, precision, and accuracy. Table 3 shows pairwise comparisons between BPNN and each of CKD-EPI, EKFC, BIS1, LMR, and Asian modified CKD-EPI, and MDRD in terms of bias and P30 values. In the whole test cohort, very similar median ratios were observed among all seven equations, but the mean square error (MSE) of BPNN was smallest (154.93) of all seven equations (Table 2). Regarding bias, the median bias of BPNN was 2.06 (0.54–3.28) ml/min/1.73 m^2^, which was significantly smaller than that of LMR (4.59 [2.95–6.17] ml/min/1.73 m^2^; p = 0.03); the median bias between BPNN and each of CKD-EPI (2.19 [1.05–3.56] ml/min/1.73 m^2^; p = 0.31), EKFC (-1.41 [-0.09−1.01] ml/min/1.73 m^2^; p = 0.26), BIS1 (0.64 [-0.09–1.01] ml/min/1.73 m^2^; p = 0.99), and MDRD (1.11 [-0.03–2.67] ml/min/1.73 m^2^; p = 0.45) was not significant (Tables 2 and 3).

**Table 2:** Bias, precision and accuracy of the 6 GFR estimation equations and BPNN model

	Median bias (95% CI)^a^	IQR (95% CI)^b^	P30 (95% CI)^c^	MSE	Median ratio^d^ (95% CI)
Test cohort (n = 244)					
CKD-EPI	-0.15 (-3.35−3.11)	18.44 (15.62−20.79)	64.75 (58.72−70.79)	234.82	0.996 (0.94−1.07)
EKFC	3.45 (1.87−5.54)	15.08 (12.27−18.12)	68.44 (62.57−74.31)	183.11	1.08 (1.03−1.14)
BIS1	2.13 (0.86−3.58)	15.18 (12.78−17.16)	74.18 (68.65−79.71)	168.61	1.05 (1.02−1.07)
LMR	4.59 (2.95−6.17)	14.74 (12.36−17.27)	67.21 (61.28−73.14)	184.10	1.11 (1.06−1.16)
Asian modified CKD-EPI	-1.43 (-5.11−0.72)	20.42 (18.30−22.76)	62.3 (56.17−68.42)	241.40	0.97 (0.9−1.01)
MDRD	0.04 (-2.44−3.0)	19.29 (16.79−21.85)	63.93 (57.87−70.0)	289.49	1.001 (0.96−1.09)
BPNN	2.06 (0.54−3.28)	14.31 (12.07−15.86)	78.28 (73.07−83.49)	154.93	1.03 (1.01−1.07)
Patients with GFR ≥ 45 ml/min/1.73 m^2^ (n = 128)					
CKD-EPI	-3.68 (-6.8− -1.12)	18.63 (15.19−22.8)	82.81 (76.19−89.44)	279.03	0.94 (0.92−0.99)
EKFC	2.75 (0.73−6.6)	14.59 (11.25−19.0)	85.94 (79.83−92.04)	215.91	1.04 (1.01−1.1)
BIS1	4.24 (2.28−6.74)	14.7 (10.65−17.76)	85.16 (78.91−91.4)	218.67	1.06 (1.04−1.11)
LMR	4.14 (1.85−7.2)	14.74 (11.74−18.46)	84.37 (77.99−90.75)	219.58	1.06 (1.03−1.13)
Asian modified CKD-EPI	-6.38 (-10.17− -2.79)	19.27 (15.8−22.49)	78.91 (71.74−86.07)	274.03	0.9 (0.88−0.97)
MDRD	-2.63 (-5.34−0.6)	21.26 (17.29−26.52)	80.47 (73.51−87.43)	369.46	0.96 (0.93−1.01)
BPNN	2.88 (1.14−5.55)	16.22 (13.86−19.38)	85.16 (78.91−91.4)	198.85	1.04 (1.02−1.08)
Patients with GFR < 45 ml/min/1.73 m^2^ (n = 116)					
CKD-EPI	4.05 (0.38−6.05)	17.04 (13.29−22.3)	44.83 (35.64−54.01)	186.05	1.16 (1.01−1.32)
EKFC	4.23 (1.14−6.5)	14.87 (11.28−18.49)	49.14 (39.9−58.37)	146.91	1.2 (1.04−1.31)
BIS1	0.24 (-2.58−1.61)	12.85 (10.3−16.29)	62.07 (53.11−71.03)	113.38	1.02 (0.94−1.07)
LMR	4.95 (2.48−7.56)	15.22 (11.63−18.57)	48.27 (39.05−57.51)	144.96	1.27 (1.1−1.41)
Asian modified CKD-EPI	2.92 (-1.33−5.1)	17.73 (13.26−22.33)	43.97 (34.8−53.13)	205.39	1.1 (0.96−1.25)
MDRD	3.49 (-1.27−5.68)	16.61 (12.95−21.06)	45.69 (36.49−54.89)	201.25	1.13 (0.96−1.28)
BPNN	0.74 (-1.55−2.78)	12.46 (9.98−14.65)	70.69 (62.28−79.1)	106.48	1.02 (0.93−1.145)

CKD-EPI, Chronic Kidney Disease-Epidemiology equation; EKFC, European Kidney Function Consortium equation; BIS1, Berlin Initiative Study-1 equation; LMR, Lund-Malmö Revised equation; BPNN, Back propagation neural network model; MDRD, Modification of Diet in Renal Disease equation; GFR, glomerular filtration rate; CI, confidence interval; MSE, mean square error. The 95% CIs were calculated by a bootstrap method (1000 bootstraps) over all measure differences

^a^Bias was the median difference between measured GFR by ^99m^Tc-DTPA renal dynamic imaging and estimated GFR by a given formular; a negative bias means that, on average, an equation overestimates the GFR.

^b^Precision was the interquartile range (IQR) of the differences between measured GFR and estimated GFR; the smaller the IQR of the difference, the greater precision

^c^P30 represents the percentage of estimated GFR within 30% of measured GFR; the higher the P30, the greater accuracy

^d^The ratio referred to measured GFR divided by estimated GFR; less than 1 means that, on average, an equation overestimates the GFR.

**Table 3:** Pairwise comparisons between BPNN and other GFR estimation equations

Pairwise comparisons	Difference in bias^a^	p	Difference in P30^b^	p
	Median (95% CI)		Median (95% CI)	
BPNN–CKD-EPI	2.19 (1.05 to 3.56)	0.31	13.52 (5.61 to 21.44)	< 0.001
BPNN–EKFC	-1.41 (-0.09 to 1.01)	0.26	9.84 (2.04 to 17.63)	0.008
BPNN–BIS1	0.64 (-0.09 to 1.01)	0.99	4.10 (-3.45 to 11.64)	0.98
BPNN–LMR	-2.42 (-2.86 to -1.93)	0.03	11.07 (3.22 to 18.91)	0.001
BPNN–Asian modified CKD-EPI	4.75 (3.78 to 6.32)	0.02	15.98 (8.0 to 23.97)	< 0.001
BPNN–MDRD	1.11 (-0.03 to 2.67)	0.45	14.34 (6.4 to 22.29)	< 0.001

GFR, glomerular filtration rate; CI, confidence interval; CKD-EPI, Chronic Kidney Disease-Epidemiology equation; EKFC, European Kidney Function Consortium equation; BIS1, Berlin Initiative Study-1 equation; LMR, Lund-Malmö Revised equation; BPNN, Back propagation neural network model; MDRD, Modification of Diet in Renal Disease equation. The 95% CIs were calculated by a bootstrap method (1000 bootstraps) over all measure differences

^a^Median difference in bias is the difference between equation biases (measured GFR minus estimated GFR), and a positive median difference means that, on average, the second equation provides higher values of estimated GFR than the first one

^b^P30 represents the percentage of estimated GFR within 30% of measured GFR; a positive median difference means that, on average, more values from the first equation are close to measured GFR.

Regarding precision, the BPNN tool was the most accurate; it had the highest precision: 14.31 (12.07–15.86) ml/min/1.73 m^2^ (Table 2). The precision IQRs of CKD-EPI, EKFC, BIS1, and LMR equations were 18.44 (15.62–20.79) ml/min/1.73 m^2^, 15.08 (12.27–18.12) ml/min/1.73 m^2^, 15.18 (12.78–17.16) ml/min/1.73 m^2^, and 14.74 (12.36–17.27) ml/min/1.73 m^2^, respectively. The precision IQRs of the new Asian modified CKD-EPI and MDRD were 20.42 (18.30−22.76) and 19.29 (16.79−21.85), respectively. Regarding accuracy, the P30 of BPNN was highest: 78.28% (73.07–83.49%). The P30 between BPNN and each of CKD-EPI, EKFC, and LMR was significant (more accurate in the former) (13.52%, p < 0.001; 9.84%, p = 0.008, and 11.07%, p = 0.001; respectively). The P30 between BPNN and each of the new Asian modified CKD-EPI and MDRD was also significant (15.98%, p < 0.001; 14.34%, p < 0.001). The P30 of BPNN vs. BIS1 was not significant (4.1% [-3.45–1.64%]; p = 0.98) (Table 3).

### Quantile regression

**Fig. 2:**
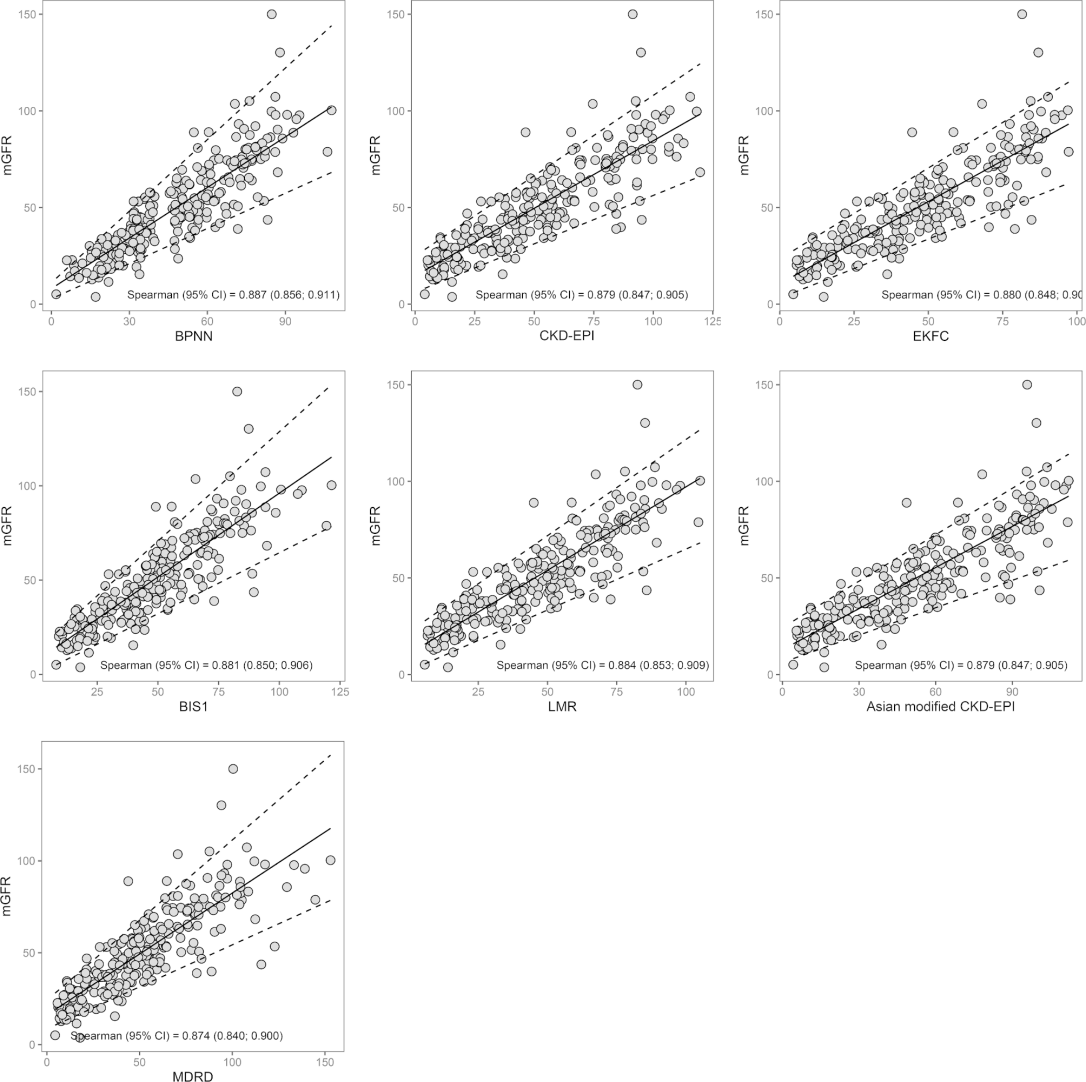
Quantile regression graphs evaluating the correlation between estimated GFR and GFR measured by ^99m^Tc-DTPA renal dynamic imaging (mGFR). The solid lines indicate the regression line and the dashed lines indicate the 0.05 and 0.95 quantile. GFR, glomerular filtration rate; ^99m^Tc-DTPA, technetium-99 m-diethylene triamine pentaacetic acid; CKD-EPI, Chronic Kidney Disease-Epidemiology equation; EKFC, European Kidney Function Consortium equation; BIS1, Berlin Initiative Study-1 equation; LMR, Lund-Malmö Revised equation; BPNN, Back propagation neural network model; MDRD, Modification of Diet in Renal Disease equation

The quantile regression graphs demonstrate good correlation between the measured GFR and the BPNN, CKD-EPI, EKFC, BIS1, LMR, Asian modified CKD-EPI, and MDRD equations (Fig. 2). In the Spearman’s correlation, there were no significant differences between the equations and the measured GFR, suggesting robust correlation values in all assessments. BPNN has the highest regression coefficient (0.89).

### Concordance correlation coefficient

Statistically, the strength of agreement between two methods can be assessed by the Lin’s CCC (Figure S1). In the test population, a CCC of 0.86 (95% CI 0.82−0.89) was calculated for BPNN, thereby indicating that there is relatively good agreement. The CCCs for BIS1, Asian modified CKD-EPI, and MDRD were 0.85 (95% CI 0.81−0.88), 0.83 (95% CI 0.79−0.87), and 0.81 (95% CI 0.77−0.85), respectively.

### Subgroup analysis

We then did subgroup analysis between patients with less than 45 and those with greater than 45 ml/min/1.73 m^2^. At measured GFR < 45 ml/min/1.73 m^2^, the BPNN has the lowest MSE (106.48), highest accuracy P30 (70.69%), and highest precision IQR (12.46). The biases of BPNN and BIS1 equations were similar (0.74 [-1.55−2.78] and 0.24 [-2.58−1.61], respectively), which were smaller than any other equation.

## Discussion

We developed a new equation, the BPNN model that combines the four variables of age, sex, serum creatinine, and diabetes, to estimate GFR in persons aged 65 years and older. Using data from an independent test cohort of 244 individuals, we validated the BPNN model and showed that it was more accurate than the widely-used CKD-EPI equation as well as the EKFC, BIS1, LMR, and MDRD equations. The BPNN model has highest precision and greatest accuracy among aforementioned equations, although the bias is not optimal. This has important implications for public health and clinical practice.

Machine learning is an emerging field of medicine where vast resources are applied to integrate computer science and statistics into medical problems [24]; it can assemble large clinical databases, and generate tools for decision-making in various areas of human health [25]. Deep machine learning, such as BPNN, can learn complex nonlinear relationships between heterogeneous kinds of data and has the advantage of detecting all possible interactions between the predictor variables [26]. Actually, we developed four machine learning models (random forest, support vector machine, classification and regression tree, and BPNN) and compared their predictability. According to the MAE and R squared, the BPNN had the relative superiority over three other machine learning models.

As with the previously established GFR-estimating equations, we also included age and sex [6–9]. This is because these parameters correlated with muscle mass, which is the main determinant of creatinine generation, albeit these parameters do not account for all variation in non-GFR determinants of serum creatinine. Given the possible effects of diabetes [11, 27, 28], this study only integrated four variables of specific interest (i.e., age, sex, serum creatinine and diabetes status), and found that deep machine learning such as BPNN could achieve a superior GFR-estimating model in an older population. The greater accuracy of the BPNN model could improve clinical decision-making in older patients with decreased renal function. This is because early referral and treatment of CKD in patients, especially in elderly patients, may reduce mortality, hospitalization rates, and dialysis catheter use.

The MDRD equation, which was developed in whites and African Americans with CKD, tends to have differences in performance among subgroups [4]. For this reason, a new equation, the CKD-EPI equation was established from adults of any age in North America and Europe with and without kidney function loss; the proportion of patients aged ≥ 65 years within the equation development and validation datasets was only 13%.^2^ Previous studies have found that the CKD-EPI equation had adequate performance in older populations with different levels of GFR [29]. The Kidney Disease Improving Global Outcomes (KDIGO) guidelines also recommend the CKD-EPI creatinine equation is preferred in adult patients [1]. However, further improvement seems to be required at GFR < 60 ml/min/1.73 m^2^ [11, 30].

In addition, although the original CKD-EPI equation takes four-level race (Black, Asian, Native American and Hispanic and others) into account, it requires more research to validate the performance of this equation in Chinese patients. Therefore, a new Asian modified CKD-EPI equation was developed aiming to improve the performance of the original one in determining GFR in Chinese adults with CKD [5]. However, in the present, we did not find superiority of this equation. This may be due to differences in the age of inclusion, and the different degrees of kidney function loss.

The EKFC equation is a modified FAS creatinine-based equation that combines the properties of the FAS and CKD-EPI equations and can be applied to the full spectrum of age and kidney function [9]. It was predominantly developed in a multicentre study in a predominantly European population and was proposed to address the problem of overestimation of GFR in the young and old. In this study, the EKFC equation is not optimal when compared to the BPNN model based on the establishment of an older population.

There are few studies specifically to develop GFR-estimating equations in older adults. The LMR equation was developed in a cohort of 850 Swedish Caucasians referred for GFR measurement [6]. In this population, approximately half of the participants were over 60 years old. In settings similar to the study cohort, the performance of LMR over CKD-EPI was inconsistent in terms of bias, precision and accuracy [31, 32]. In external validation of older Chinese populations, there also did not appear to be a performance advantage for the use of the LMR Eqs. [11, 33]. In the present study, the performance of the BPNN model was not only superior to that of CKD-EPI but also superior to that of LMR.

The BIS1 equation was developed in a population-based cohort of 570 patients aged 70 years and older who underwent iohexol clearance measurement [7]. Compared to iohexol clearance, BIS1 had excellent performance for GFR estimation in this age group (median bias, 0.8 ml/min/1.73 m^2^; precision IQR, 11.1 ml/min/1.73 m^2^; P30, 95%). Other studies, however, had some inconsistent results [8, 33–36]. In our study, the performance of BIS1 was second to that of the BPNN model in elderly individuals among the 6 creatinine-based GFR-estimating equations.

All the performance above revealed the BPNN model was more accurate than other equations in this age group. These results are consistent with previous results from the validation dataset [10]. Specifically, the advantage of the BPNN model was mainly reflected in lower GFR group. This can help improve the management of CKD. In this study, we used ^99m^Tc-DTPA renal dynamic imaging as a reference method for GFR measurement. To date, measurements of GFR have generally relied on renal clearance of exogenous filter markers (e.g., inulin, iohexanol, and ^99m^Tc-DTPA) [37] or endogenous filter markers such as creatinine and cystatin C [38]. ^99m^Tc-DTPA renal dynamic imaging for measuring GFR, which is recommended by the Nephrology Committee of the Society of Nuclear Medicine [39], is widely used in daily clinical work [40, 41].

The study had several limitations, such as a lack of cystatin C, which is less affected by muscle mass. More accurate GFR measurements, such as inulin clearance, were not applied, as inulin is typically used in research work and is too inconvenient for use in everyday practice. Additionally, the sample size of the test dataset is relatively small. Further studies with a larger older population are required to externally validate the performance of our novel model. Third, as this was a retrospective study, we could not calculate the bias when mixing a steady state infusion method with a true clearance calculation such as ^99m^Tc-DTPA and a single shot plasma disappearance. Nevertheless, its renal clearance has been found to be close to inulin, with a consistency of 0.99 over a wide range of GFR, suggesting that the renal system treats these similarly [42]. Finally, the novel model does not overcome the limitations of serum creatinine as an endogenous filtering marker. However, as creatinine is currently routinely measured and is central to the clinical assessment of renal function, serum creatinine-based GFR estimates will continue to be used in actual clinical practice in the foreseeable future.

## Conclusion

In summary, the machine learning method (i.e., BPNN) improved the precision and accuracy of methodologies for estimating GFR, although bias remains suboptimal. The 4-variable novel BPNN tool was more accurate than the currently available creatinine-based GFR estimation equations in elderly individuals, especially in older adults with GFR below 45 ml/min/1.73 m^2^. Based on deep machine learning, future research should be directed towards the evaluation of cystatin C for GFR estimation, either alone or in combination with serum creatinine.

## Electronic supplementary material

Below is the link to the electronic supplementary material.

**Additional file 1:** Table S1.

**Additional file 1:** Table S2.

**Additional file 3:** Figure S1.

## Data Availability

All relevant data are disclosed in the manuscript and its associated figures and Supplementary Materials. Further inquire related to the study should be directed to the corresponding author.

## Abbreviations

GFR: glomerular filtration rate
BPNN: Back propagation neural network
CKD-EPI: Chronic Kidney Disease-Epidemiology Collaboration
EKFC: European Kidney Function Consortium
BIS1: Berlin Initiative Study-1
LMR: Lund-Malmö Revised
MDRD: Modification of Diet in Renal Disease
CKD: chronic kidney disease
^99m^Tc-DTPA: technetium-99 m-diethylene triamine pentaacetic acid
BSA: body surface area
SD: standard deviation
IQR: interquartile range
CI: confidence interval.
