# Supplementary material for: A back propagation neural network approach to estimate the glomerular filtration rate in an older population
Source: BMC Geriatr. 2023 May 24;23:322. doi: 10.1186/s12877-023-04027-5 (PMC10207816; doi:10.1186/s12877-023-04027-5)
Supplement: Supplementary file 1 — Additional file 1: Table S1. [file 12877_2023_4027_MOESM1_ESM.docx]

**Supplementary file**

**Table S1**. Six creatinine-based equations for estimating glomerular filtration rate.

| **EKFC (European Kidney Function Consortium) equation**  Scr/Q<1 107.3 × (Scr/Q)^-0.322^ × 0.990^(Age − 40)^  ≥1 107.3 × (Scr/Q)^-1.132^ × 0.990^(Age − 40)^  Q values, for ages >25 years:  Males: Q=80 μmol/L  Females: Q=62 μmol/L |
| --- |
| **CKD-EPI (Chronic Kidney Disease Epidemiology Collaboration) equation**  141 × min(Scr/κ, 1)^α^× max(Scr/κ, 1)^-1.209^ × 0.993^Age (years)^ (×1.018 if female)  κ is 0.7 for females and 0.9 for males; α is -0.329 for females and -0.411 for males, min indicates the minimum of Scr/κ or 1, and max indicates the maximum of Scr/κ or 1. Scr in mg/dL. |
| **BIS1(Berlin Initiative Study-1) equation**  3736 × Scr^-0.87^ × Age^-0.95^ × 0.82 (if female)  Scr in mg/dL and age in year |
| **LMR (Lund-Malmö Revised) equation**  =e^X-0.0158×Age+0.438×ln(Age)^  Female and Scr <150 μmol/L: X=2.50 + 0.0121 × (150 – Scr)  Female and Scr ≥150μmol/L: X=2.50 – 0.926 × ln(Scr/150)  Male and Scr <180μmol/L: X=2.56 + 0.00968 × (180 – Scr)  Male and Scr ≥180μmol/L: X=2.56 – 0.926 × ln(Scr/180) |
| **MDRD (Modification of Diet in Renal Disease Study) equation**  175×Scr[mg/dl]^-1.154^×age[years]^-0.203^×0.742[if female] |
| **Asian modified CKD-EPI**  Female and Scr≤0.7mg/dl: 151×(Scr/0.7)^-0.328^×0.993^age^  Female and Scr>0.7mg/dl: 151×(Scr/0.7)^-1.21^×0.993^age^  Male and Scr≤0.9mg/dl: 149×(Scr/0.9)^-0.415^×0.993^age^  Male and Scr>0.9mg/dl: 149×(Scr/0.7)^-1.21^×0.993^age^  Scr in mg/dL and age in year |

Scr, serum creatinine.
