# Supplementary material for: A back propagation neural network approach to estimate the glomerular filtration rate in an older population
Source: BMC Geriatr. 2023 May 24;23:322. doi: 10.1186/s12877-023-04027-5 (PMC10207816; doi:10.1186/s12877-023-04027-5)
Supplement: Supplementary file 3 — Additional file 3: Figure S1. [file 12877_2023_4027_MOESM3_ESM.docx]

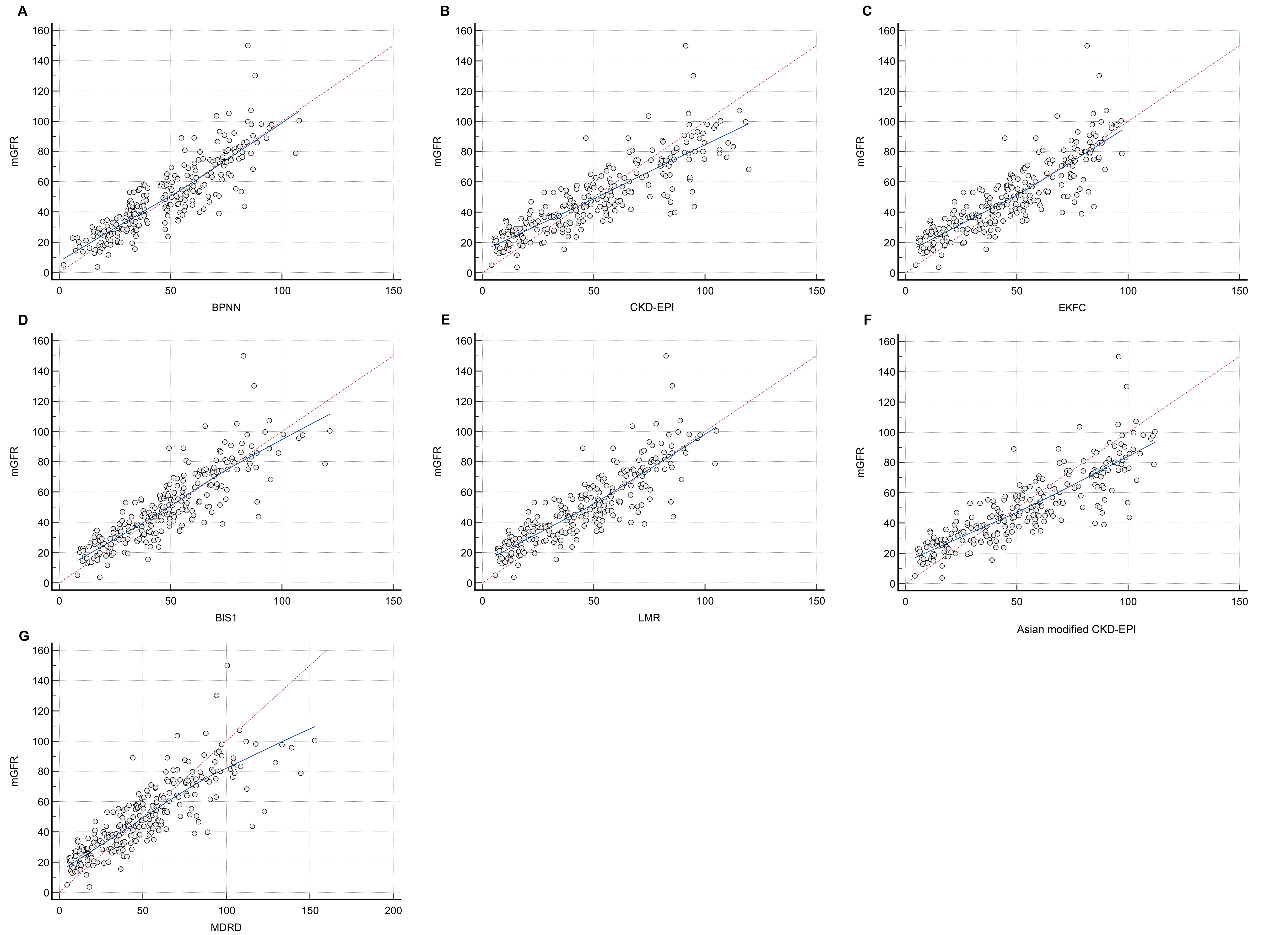


**Figure S1**. Lin’s concordance correlation coefficient (CCC) between estimated GFR and GFR measured by ^99m^Tc-DTPA renal dynamic imaging (mGFR). The CCC for panel A is 0.86 (95% CI, 0.82−0.89); B, 0.84 (95% CI, 0.79−0.87); C, 0.85 (95% CI, 0.81−0.88); D, 0.85 (95% CI, 0.81−0.88); E, 0.85 (95% CI, 0.81−0.88); F, 0.83 (95% CI, 0.79−0.87); G, 0.81 (95% CI, 0.77−0.85). GFR, glomerular filtration rate; ^99m^Tc-DTPA, technetium-99 m-diethylene triamine pentaacetic acid; CKD-EPI, Chronic Kidney Disease-Epidemiology equation; EKFC, European Kidney Function Consortium equation; BIS1, Berlin Initiative Study-1 equation; LMR, Lund-Malmö Revised equation; BPNN, Back Propagation neural network model; MDRD, Modification of Diet in Renal Disease equation. Graphs of CCC were created with MedCalc (version 20.0.15; MedCalc, Mariekerke, Belgium).
